# The estimation of patients' views on organizational aspects of a general dental practice by general dental practitioners: a survey study

**DOI:** 10.1186/1472-6963-11-263

**Published:** 2011-10-11

**Authors:** Rutger E Sonneveld, Michel Wensing, Ewald M Bronkhorst, Gert-Jan Truin, Wolter G Brands

**Affiliations:** 1 Department of Preventive and Restorative Dentistry, Radboud University Nijmegen Medical Centre, Philips van Leijdenlaan 25, 6525 EX Nijmegen, the Netherlands; 2 Scientific Institute for Quality of Healthcare, Radboud University Nijmegen Medical Centre, Geert Grooteplein 21, 6525 EZ Nijmegen, the Netherlands

## Abstract

**Background:**

Considering the changes in dental healthcare, such as the increasing assertiveness of patients, the introduction of new dental professionals, and regulated competition, it becomes more important that general dental practitioners (GDPs) take patients' views into account. The aim of the study was to compare patients' views on organizational aspects of general dental practices with those of GDPs and with GDPs' estimation of patients' views.

**Methods:**

In a survey study, patients and GDPs provided their views on organizational aspects of a general dental practice. In a second, separate survey, GDPs were invited to estimate patients' views on 22 organizational aspects of a general dental practice.

**Results:**

For 4 of the 22 aspects, patients and GDPs had the same views, and GDPs estimated patients' views reasonably well: 'Dutch-speaking GDP', 'guarantee on treatment', 'treatment by the same GDP', and 'reminder of routine oral examination'. For 2 aspects ('quality assessment' and 'accessibility for disabled patients') patients and GDPs had the same standards, although the GDPs underestimated the patients' standards. Patients had higher standards than GDPs for 7 aspects and lower standards than GDPs for 8 aspects.

**Conclusion:**

On most aspects GDPs and patient have different views, except for social desirable aspects. Given the increasing assertiveness of patients, it is startling the GDP's estimated only half of the patients' views correctly. The findings of the study can assist GDPs in adapting their organizational services to better meet the preferences of their patients and to improve the communication towards patients.

## Background

In the Netherlands, general dentistry is a healthcare sector which is comparable in size to primary medical care in terms of costs [1] and numbers of patients [2,3]. While much research has been conducted concerning the organization of primary medical care [4-7], research with regard to organizational aspects of a general dental practice is scarce. In the Netherlands, several organizational changes in dental care can be observed in recent years. Firstly, clinical tasks are increasingly being delegated from general dental practitioners (GDPs) to other health professionals [8,9]. Secondly, the number of GDPs per dental practice continues to increase [10]. Thirdly, the structural context has changed. New health laws will be introduced [11] together with the implementation of market competition in dental healthcare. In order to respond to these changes, GDPs are legally obliged to be more transparent by providing information about the quality of their performance to support patients to make informed choices [12]. Although GDPs are supposed to meet those preferences, insight by GDPs in the preferences of patients to support decision-making is lacking [13].

In recent years, the Dutch government has launched the "Visible Care" program [14] in order to increase transparency in healthcare. The Visible Care program seeks to provide patients with (1) medical information concerning the safety, efficiency, efficacy, and patient-centeredness of healthcare, (2) information concerning organizational aspects of healthcare, such as information on opening hours and accessibility; and (3) survey patients' experiences with the healthcare delivered, measured with the Consumer Quality index (CQ-index), which is based on the American CAHPS (Consumer Assessment of Health care Providers and Systems) questionnaire and Dutch QUOTE (QUality Of care Through the patient's Eyes) instrument [15].

As part of the Visible Care Program, this study explores the views of patients and GDPs on organizational aspects of a general dental practice. Research in primary medical care showed that patients and physicians have similar views about preferences on the medical practice care and physicians could assess patients' preferences reasonably well [16]. The aim of this study was to examine whether GDPs adequately can estimate the views of patients with respect to a number of organizational aspects of a general dental practice.

## Methods

### Design

Two survey studies were combined. In the first study, a questionnaire was developed for assessing the views of patients and GDPs on a number of organizational aspects of a general dental practice. This questionnaire was developed after reviewing the research literature and was based on aspects used in the International Organization for Standardization (ISO) 9001, the Dutch HKZ model (which is comparable with ISO, translated to the Dutch health care) [17,18], and the European Practice Assessment instrument [19,20]. A set of 169 organizational aspects was initially composed. The combined list was rated with respect to usefulness and overlap. Next, several aspects were clustered at a higher aggregation level and made operational. The questionnaire was rated by participants in three focus group meetings on usefulness, relevance and clarity (two consisting of 8 and 13 patients respectively and one consisting of 11 GDPs). This resulted in a questionnaire consisting of 39 questions, containing a list 41 organizational aspects of a general dental practice. The aspects were divided into five domains: (I) infrastructure; (II) staff; (III) information; (IV) finance; and (V) quality and safety [19]. The questions had different multiple choice categories, reflecting possible standards for a specific organizational aspect. For example, respondents were asked how soon the telephone should be answered when they call a dental practice or whether they preferred a reminder for a routine oral examination. A final question was added to document the 10 most important aspects (out of the 41) for assessing a general dental practice. Two questionnaires were developed: one for patients and one for GDPs. Finally, the patient questionnaire was pilot tested among 50 patients in a general dental practice, resulting in small refinements. In the second survey study, a questionnaire was developed for GDPs based on the questionnaire in the first survey. The questions were reworded, for example: "What do you think the patient prefers?". GDPs estimated what percentage they thought the patient would give for each category. For example, a GDP estimated that 15% of the patients would have answered the first answering category; 30% the second category; 40% the third category; and 15% the last answering category. In total, the answers added up to 100%. To avoid large time constraints when filling in the questionnaire, in the second study, we decided to only ask the GDPs about the top 20 aspects from the first study, resulting in 22 questions: the aspect 'making appointments' was divided into 3 types of appointments, see additional file 1: questionnaire GDPs' estimation of patients. To summarize, each question was asked 3 times: to patients; to GDPs; and, in reworded format, to GDPs who give their estimation of the patients' views.

### Study populations

In the first study, the questionnaire was sent to 5000 dental patients divided over 100 general dental practices, which had been selected at random. Each GDP handed out the questionnaires to the first 50 patients visiting the practice during the assigned period. After 2 weeks, the GDPs sent a reminder to these patients. The GDPs were asked to fill in the GDP questionnaire as well. In addition to the GDPs participating in the patient study, a representative sample of 400 GDPs was sent a questionnaire. If the GDPs did not respond, a reminder was sent after 3 weeks and if there was still no response after 5 weeks, the questionnaire was sent again.

In the second study, a representative sample of another 400 Dutch GDPs was drawn (GDPs participating in the first study were excluded from the second study). The reminder procedure for non-responders was the same as that used in the first study.

Because no medical data or personal information of respondents was used, the study did not need an ethical approval.

### Analyses

The results of the first and second survey were combined for analyses. Frequency distributions were calculated using the statistical package SPSS, version 16.0.

In this article, we focus on differences in views of patients and GDPs' estimation of patients' views. Difference between patient views and the estimation by GDPs of these views, can be either towards the views of the GDPs themselves, or in the opposite direction. To allow this analysis, the views of GDPs, although not subject of study here, are presented as well.

For the analyses of the GDPs' estimation of patients' views, we categorized the findings as follows: (1) GDPs estimated the patients' views well and the patients' views were similar to those of the GDPs; (2) GDPs estimated the patients' views well, but the patients' views differed from those of the GDPs; (3) GDPs estimated the patients' views poorly, but the patients' views were the same as those of the GDPs; and (4) GDPs estimated the patients' views poorly and the patients' views differed from those of the GDPs.

For the estimation, we examined the distribution of the answering categories of the GDPs' estimation of patients' views and compared this with the answering categories of the patients and GDPs. If an answering category of patients' views and GDPs' estimation of patients' views differed by more than 10%, we concluded the aspect to be answered differently.

In case the aspect was judged differently, we compared the answers given by analyzing at the stringency of preferences. We presume that GDPs overestimate the views of the patients if the GDPs tend to expect patients to select answering categories which demand a larger effort of a dental practice. For example: longer opening hours, shorter waiting times, or longer continuing education.

## Results

Table 1 shows the characteristics of the patient sample and the samples of GDPs in the first and second study compared with Dutch national data. Among the patients, the response rate was 63% (*n *= 3127); 41% was male and 59% was female. The largest group was aged 40-64 years (60%).

**Table 1:** Characteristics of patients and GDPs in the first and second study compared with Dutch national data

		Patients	National data patients	1^st ^study GDPs	2^nd ^study GPDs	National data GPDs
Gender						
	Male	41.1	47.4	72.8	66.1	69.1
	Female	58.9	52.6	27.2	33.9	30.9
						
Age (years)						
	< 20	1.3	5.9	0.0	0.0	0.0
	20-39	23.7	31.2	22.1	30.0	29.2
	40-64	60.0	44.1	76.8	68.2	70.7
	> 65	15.0	18.8	1.0	1.8	0.1

The response rate of the GDPs was 61% in the first study and 30% in the second study. 73% and 66% of the GDPs were male in the first and second study, respectively. The age distribution of GDPs in both samples did not differ significantly compared with national data.

Table 2 shows the results of the study. For the majority of the aspects, the respondents mentioned the same answering category the most. For 5 aspects, patients and GDPs did not mention the same answering category the most (in the domain *infrastructure*: 'availability of an appointment for a routine oral examination', 'practice accessibility', and 'parking spaces'; in the domain *staff*: 'specialties in dental practice'; and in the domain *quality and safety*: 'protocols and guidelines'), and for 4 aspects, patients had a different highest answering category than the GDPs' estimation of patients' views (in the domain *infrastructure*: 'practice accessibility' and 'opening hours in the evening and/or weekend'; in the domain *staff*: 'specialties in dental practice'; and in the domain *quality and safety*: 'quality assessment').

**Table 2:** Distribution (%) of the answers on the organizational aspects of a general dental practice given by patients, by GDPs' estimation of patients' views, and by GDPs

Rank (domain)	Aspect	Patients	GDPs' estima- tion	GDPs	Rank (domain)	Aspect	Patients	GDPs' estima- tion	GDPs
**1**	**Accessibility by telephone**				**9**	**Check-up of perishable goods**			
**(I)**	directly	5.1	10.5	2.0	**(V)**	yes	97.0	82.4	83.8
	within 15 sec	20.6	25.7	32.9		does not matter	2.5	13.3	8.9
	15-30 sec	30.1	37.0	36.5		no	0.5	4.2	7.3
	30-60 sec	28.9	18.2	21.3	**10**	**Treatment by same dental therapist**			
	more than 60 sec	4.5	3.5	3.3	**(II)**	by the same person	74.2	67.7	68.1
	does not matter	10.8	5.1	4.0		by someone with the same education	8.9	14.3	4.4
**2**	**Continuing education dentist**					according to same treatment plan	10,5	10.0	21,8
**(II)**	yes, 0-8 hours	5.4	7.9	4.3		does not matter	5.5	5.1	3.4
	yes, 8-24 hours	17.5	19.1	29.8		no	1.0	2.9	2.3
	yes, 24-40 hours	10.6	22.0	21.2	**11**	**Specialties in dental practice**			
	yes, more than 40	3.5	11.6	6.6	**(II)**	yes	41.1	37.9	22.3
	yes, but any length is ok	62.4	36.8	37.7		does not matter	40.0	42.8	29.9
	no	0.7	2.6	0.3		no	18.9	19.3	47.8
**3**	**Dutch-speaking dentists**				**12**	**Information on tasks of staff**			
**(V)**	yes	97.7	91.3	98.7	**(V)**	yes	70.8	83.3	89.4
	does not matter	2.2	7.3	1.0		does not matter	26.2	13.5	10.0
	no	0.1	1.4	0.3		no	3.0	3.2	0.7
**4**	**In office waiting times**				**13**	**Working according to professional standards**			
**(I)**	none	1.4	9.0	2.3	**(V)**	yes	58.0	64.9	82.1
	1-5 min	18.5	28.1	22.2		what is a professional standard?	41.5	33.7	17.5
	6-10 min	48.3	33.0	34.4		no	0.6	1.4	0.3
	11-15 min	25.5	19.9	31.5	**14**	Information on dental bill*			
	16-20 min	5.9	8.3	8.6	**(III)**	treatment	95.2	80.3	95.4
	more than 20 min	0.5	1.6	1.0		date	76.4	73.3	96.0
**5**	Information about dental services*					amount	85.9	85.0	96,7
**(III)**	written	48.0	42.2	72.9		payment terms	47.9	48.3	91.4
	internet	37.2	34.8	41.9		name dental professional	38.8	30.4	51.8
	oral	48.7	51.3	80.5	**15**	**Reminder of routine oral examination**			
	does not matter	18.2	15.0	11.6	**(III)**	yes	61.4	61.8	58.9
**6.1**	**Availability of appointments (waiting lists)**					does not matter	20,5	23,4	17.8
**(I)**	***routine oral examination***					no	18.1	14.8	23.2
	directly	0.4	3.2	0.0	**16**	**Opening hours in the evening and/or weekends**			
	same day	1.0	3.9	2.3	**(I)**	only in the evening	15.2	19.2	3.7
	within 2 days	4.4	6.9	1.3		only in the weekend	5.5	8.9	0.7
	within 1-2 weeks	42.4	55.5	36.4		evening and weekend	18.4	31.0	4.7
	within 2-4 week	40.7	24.5	52.3		does not matter	16.5	17.8	7.0
	longer than 4 weeks	11.1	6.0	7.6		no	44.4	23.1	84.0
**6.2**	***broken tooth***				**17**	**Practice accessibility**			
	directly	6.5	1.8	1.7	**(I)**	less than 2 km	14.3	16.6	1.0
	same day	26.9	9.7	11.6		2-5 km	39.9	28.7	11.3
	within 2 days	33.7	47.6	51.3		5-10 km	27.4	32.8	20.0
	within 1-2 weeks	30.3	35.4	32.1		more than 10 km	2.9	7.1	7.7
	within 2-4 week	1.7	4.8	3.3		does not matter	15.5	14.9	60.0
	longer than 4 weeks	0.8	0.7	0.0	**18**	**Accessibility for disabled patients**			
**6.3**	***pain complaints***				**(I)**	yes	88.2	66.3	86.8
	directly	18.4	23.0	14.2		does not matter	9.3	23.3	6.6
	same day	61.3	60.4	78.5		no	2.5	10.4	6.6
	within 2 days	15.0	16.2	6.0	**19**	**Parking spaces**			
	within 1-2 weeks	3.7	0.4	0.3	**(I)**	does not matter	23.9	26.3	13.6
	within 2-4 week	0.6	0.0	1.0		1-2 places	23.0	32.3	51.5
	longer than 4 weeks	1.0	0.0	0.0		more than 3	53.1	41.4	34.9
**7**	Guarantee*				**20**	**Working according protocols and guidelines**			
**(IV)**	filling	61.4	63.5	64.4	**(V)**	yes, always	52.7	n/a	33.0
	crown	80.4	72.9	69.3		yes, but diverge considered	29.4	n/a	60.3
	prosthesis	69.5	66.6	57.4		does not matter	3.8	n/a	2.0
	does not matter	7.8	9.1	12.5		unfamiliar with protocols and guidelines	13.6	n/a	2.7
	no	2.2	3.4	16.5		no	0.6	n/a	2.0
**8**	**Quality assessment**								
**(V)**	once	2.9	11.7	4.5					
	every 6 months	6.3	10.8	0.7					
	every year	36.8	31.1	9.2					
	every 2 years	47.7	30.6	45.2					
	does not matter	4.5	14.0	21.6					
	no	1.8	1.8	18.8					

Domains: I, infrastructure; II, staff; III, information; IV, finance; V, quality and safety;

* More answers are possible

Based on the outcomes presented in Table 2, Table 3 shows a summary of the GDPs' estimations compared to the views of patients. For only 4 aspects, there was consensus between the views of patients, the views of GDPs and GDPs' estimated views of patients. For 4 aspects, the GDPs' estimation of patients' views was correct; however, the GDPs set higher standards than the patients. For only 1 aspect the GDPs underestimated the patients' views ('accessibility for disabled patients') while the views of patients and GDPs were similar; GDPs believed patients to have less strict standards than is the case: GDPs underestimated patients' views on 4 aspects ('practice accessibility', 'parking spaces', 'check-up perishable goods', and 'quality assessment') while the patients had higher standards than the GDPs and underestimated on 1 aspect ('availability of an appointment for a broken tooth') while the patients had lower standards than GDPs. GDPs overestimated patients on 6 aspects ('availability of an appointment for routine oral examination', 'in-office waiting times', 'opening hours, accessibility by telephone', continuing education GDP', and 'information on tasks of staff').

**Table 3:** GDPs' estimation of patients' standards, compared to the standards of patients and GDPs

	GDPs estimated patients' standards well	GDPs underestimated patients' standards	GDPs overestimated patients' standards
GDPs and patients had the **same **standards	Dutch-speaking GDP	Accessibility for disabled patients	-
	Guarantee		
	Treatment by same GDP		
	Reminder routine oral examination		
GDPs had **lower **standards than patients	Specialties in dental practice	Practice accessibility	Availability of an appointment for routine oral examination
		Parking spaces	In-office waiting times
		Check-up of perishable goods	Opening hours
		Quality assessment	
GDPs had **higher **standards than patients	Information about dental services	Availability of an appointment for a broken tooth	Accessibility by telephone
	Availability of an appointment for pain complaints		Continuing education GDP
	Professional standards		Information on tasks of staff
	Information on dental bill		

## Discussion

In this study, we examined the GDPs' estimation of the patients' views compared to the views of patients and GDPs on a number of organizational aspects of a general dental practice.

The response rates of the first study were reasonably good: 63% for patients and 61% for GDPs. In this study the results of two GDP samples were combined. Both samples were drawn randomly from the Dutch general dental practitioners' population. Although these samples were drawn separately, we assume that the combined results of both samples represent even better the views of Dutch GDPs on organizational aspects of general dental practices.

Compared to the first survey, the response rate in the second study was low (30%). However, a comparison of the two samples on gender and age distribution with national figures indicated a good representation of the GDPs in both studies. Nevertheless, the findings should be interpreted cautiously.

For 4 out of 22 aspects, patients and GDPs had the same views and GDPs estimated patients' views reasonably well ('Dutch-speaking GDP', guarantee on treatment, 'treatment by same GDP' and 'reminder about routine oral examination'). For one aspect ('accessibility for disabled patients') patients and GDPs had the same views, but GDPs underestimated patients' views. Patients had higher standards than GDPs for 8 aspects, of which only one aspect was well estimated, and lower standards than GDPs for 8 aspects, of which 4 aspects were well estimated. In total, 9 aspects were variably well estimated by GDPs.

A correct estimation of the patients' views by GDPs was found mostly with aspects that have 'obvious' outcomes of aspects ('guarantee on treatment'); clear perception of the aspect for patients ('Dutch-speaking GDP', 'availability of an appointment for pain complaints'); and socially desirable answers ('working according to professional standards'). This also applied to the aspects concerning information. Information via the internet was the least popular answering category for patients, GDPs and the GDPs' estimation of patients' views. Although comparable health information on the internet is scarce [21], use of the internet will increase in the future [22]. It is therefore recommended that health-care providers guide patients in their internet search [23]. Regarding the information on the dental bill, it was remarkable that almost one third of the GDPs believed patients expected to see the name of the dental professional on the dental bill. An explanation could be that the patient-GDP relationship is mostly a long-term relationship, and over the last 5 years, an average of 85% of the Dutch population has visited a GDP every year [3]. Therefore the name would not be required on the dental bill.

It was remarkable that the views of GDPs and patients, and the GDPs' estimation of patients' views were approximately the same on the aspect 'guarantee on treatment'. The views of patients are understandable. Guarantee in health care is rare. In health care, physicians have an obligation to perform to the best of their ability and do not have a duty to achieve a performance. When some kind of guarantee is introduced in health care, this would have a large influence on the health law. However, in dental care, it is imaginable that GDPs could give a guarantee on some treatments that have predictable outcomes.

For the majority of the aspects, GDPs did not estimate the patients' views well. Overestimation of patients' views applied to only 6 aspects, 1 of which concerned appointment making. GDPs believed patients wanted to make an appointment as soon as possible, but patients felt differently. An explanation could be that 40% of dental patients are anxious about a dental treatment [24] and therefore want to postpone a dental appointment [25].

GDPs' underestimated patients' views on aspects concerning accessibility ('accessibility for disabled patient', 'practice accessibility', and 'parking places'), and concerning quality ('quality assessment' and 'check-up of perishable goods'). GDPs estimated that patients' views were less stringent than the patients' actual views, reflecting that patients have higher standards than GDPs perceive them to have. Some of these findings were supported by a study among retail clinics in the USA. These clinics are increasingly popular because they are often at a convenient location, prices are transparent and they seem to respond to the needs of the patient [26].

### Practice implications

Considering the organizational changes in the field of dentistry, such as the upcoming market competition, the more central position of the patient in health care, and the more obliged transparency in health care, it is important for GDPs to know which organizational aspects of a general dental practice are important for patients and how they could operationalize these. This combined study gives answers to these questions, and GDPs could use this information in their general practice to organize the dental care more to meet the preferences of their patients.

As mentioned before, there are relatively large differences between the views of patients and GDPs, and the GDPs' estimation of the patients' views. For policy makers, this information could be used for the development of guidelines, within the Visible Care program for instance. The outcomes show the aspects that will have consensus or reveal potentially conflicting areas of dental care. Looking at the aspect 'treatment by same GDP' it can be concluded that GDPs estimate the views of patients well, and the views of patients and GDPs do not differ. The implementation of a guideline on that aspect will experience little resistance.

## Conclusion

On most aspects GDPs and patient have different views, except for social desirable aspects. Given the increasing assertiveness of patients, it is startling the GDP's estimated only half of the patients' views correctly. The findings of the study can assist GDPs in adapting their organizational services to meet more the preferences of their patients and in improving the communication towards patients.

## Competing interests

The authors declare that they have no competing interests.

## Authors' contributions

RS participated in the design and the implementation of the study and drafted the manuscript. WB and GJT participated in the design of the study, in the implementation of the study, and in writing the manuscript. EB and RS conducted the statistical analyses. MW participated in the interpretation of the study results and writing the manuscript. All authors read and approved the final manuscript.

## Pre-publication history

The pre-publication history for this paper can be accessed here:

http://www.biomedcentral.com/1472-6963/11/263/prepub

## Supplementary Material

Additional file 1 **Questionnaire for GDPs**. Estimation of patients. In the additional pdf-file the questionnaire can be viewed sent to 300 GDPs regarding the estimation of the views of patients on organizational aspects of a general dental practice. Click here for file
