# Supplementary material for: The estimation of patients' views on organizational aspects of a general dental practice by general dental practitioners: a survey study
Source: BMC Health Serv Res. 2011 Oct 11;11:263. doi: 10.1186/1472-6963-11-263 (PMC3204231; doi:10.1186/1472-6963-11-263)
Supplement: Additional file 1 — Questionnaire for GDPs. Estimation of patients. In the additional pdf-file the questionnaire can be viewed sent to 300 GDPs regarding the estimation of the views of patients on organizational aspects of a general dental practice. [file 1472-6963-11-263-S1.PDF]

UMC St Radboud

Questionnaire for general dental practitioners: Do you know what patients want?

Last year, a large study has been conducted among 5000 patients and 500 general dental practitioners (GDPs). The main research question was: what information concerning organizational aspects of a general dental practice do patients and GDPs prefer to assess a general dental practice?

In this follow-up study we would like to know what you would expect patients would answer in the first study. As a result we can make a comparison between the answers given by patients and your expectation. The aim of this study is to reveal similarities or differences between the groups.

We would like to ask you to make an estimation you expect *an average patient* would give on each answering category of the questions. The answering categories of each question will add up to 100%. For the questions 18-20 the percentages can exceed the 100% because more than one answer can be given.

The results of this study will be anonymous and will not be reducible to individual GDPs.

General questions about you

A What is your gender?

Male / female

B What are the four numbers of your practice zip-code?

……………………

C What was your year of graduation?

……………………

What is your estimation of the answers given by patients?

1 When you **call** a dental practice, how long should it take before the phone is **answered**?

…… % answers *directly*

…… % answers within *15 seconds*

…… % answers *5-30 seconds*

…… % answers *30-60 seconds*

…… % answers *more than 60 seconds*

…… % answers *does not matter*

2 Within how many **kilometres** do you prefer the dental practice is physically **accessible**?

…… % answers *less than 2 km*

…… % answers *2-5 km*

…… % answers *5-10 km*

…… % answers *more than 10 km*

…… % answers *does not matter*

3 Do you prefer the dental practice to be open in the **evening** and in the **weekend**?

…… % answers *yes, only at night*

…… % answers *yes, only in the weekend*

…… % answers *at night and in the weekend*

…… % answers *does not matter*

…… % answers *no*

4 What **waiting time** is acceptable when you have an appointment?

…… % answers *no waiting time*

…… % answers *1-5 minutes*

…… % answers *6-10 minutes*

…… % answers *11-15 minutes*

…… % answers *16-20 minutes*

…… % answers *more than 20 minutes*

5 Within what time should it be possible to **make an appointment** for a**:**

Routine oral examination?

…… % answers *directly*

…… % answers *the same day*

…… % answers *within 2 days*

…… % answers *within 1 week*

…… % answers *within 1-2 weeks*

…… % answers *within 2-4 weeks*

…… % answers *longer than 4 weeks*

Broken tooth (no pain)?

…… % answers *directly*

…… % answers *the same day*

…… % answers *within 2 days*

…… % answers *within 1 week*

…… % answers *within 1-2 weeks*

…… % answers *within 2-4 weeks*

…… % answers *longer than 4 weeks*

Pain complaints?

…… % answers *directly*

…… % answers *the same day*

…… % answers *within 2 days*

…… % answers *within 1 week*

…… % answers *within 1-2 weeks*

…… % answers *within 2-4 weeks*

…… % answers *longer than 4 weeks*

6 How many **parking spaces** should a dental practice have per dental worker?

…… % answers *does not matter*

…… % answers *1-2 parking spaces*

…… % answers *more than 3 parking spaces*

7 Do you bother you have to pay a **parking fee** at a dental practice?

…… % answers *yes*

…… % answers *does not matter*

…… % answers *no*

8 Do you prefer the dental practice is **accessible** for **disabled people**?

…… % answers *yes*

…… % answers *does not matter*

…… % answers *no*

9 Should the dental practice offer different **specialties** (orthodontist etc.)?

…… % answers *yes*

…… % answers *does not matter*

…… % answers *no*

10 Should it be clear in the dental practice who **executes** which **tasks**?

…… % answers *yes*

…… % answers *does not matter*

…… % answers *no*

11 Do you think it is desirable a **GDP** should take part in **continuing education**?

…… % answers *0-8 hours per year*

…… % answers *8-24 hours per year*

…… % answers *24-40 hours per year*

…… % answers *more than 40 hours*

…… % answers *yes, length does not matter*

…… % answers *no*

12 Do you prefer the treatment by the **same dental worker**?

…… % answers *yes, same person*

…… % answers *no, but someone with same education*

…… % answers *no, but according to the same treatment plan*

…… % answers *does not matter*

…… % answers *no*

13 Do you think it is preferable you receive a **reminder** for a **routine oral examination**?

…… % answers *yes*

…… % answers *does not matter*

…… % answers *no*

14 Should the dentist work according to the **professional standard**?

…… % answers *yes*

…… % answers *what is a professional standard?*

…… % answers *no*

15 Do you prefer a **Dutch-speaking** GDP?

…… % answers *yes*

…… % answers *does not matter*

…… % answers *no*

16 Should the dental practice have a system for the **check up** of **perishable goods**?

…… % answers *yes*

…… % answers *does not matter*

…… % answers *no*

17 Should the dental practice undertake a **quality assessment**?

…… % answers *yes, once*

…… % answers *yes, least once per half year*

…… % answers *yes, least once per year*

…… % answers *yes, least once per two year*

…… % answers *does not matter*

…… % answers *no*

18 Through what media should **information** about **dental services** be available?

…… % answers *written*

…… % answers *via internet*

…… % answers *oral*

…… % answers *does not matter*

19 On which treatments do you prefer a **guarantee**?

…… % answers *yes, on a filling*

…… % answers *yes, on a crown*

…… % answers *yes, on a prosthesis*

…… % answers *does not matter*

…… % answers *no*

20 What **information** should be on a **dental bill**?

…… % answers *treatment*

…… % answers *date*

…… % answers *amount*

…… % answers *payment terms*

…… % answers *name dental practitioner*

Thank you for filling in the questionnaire. We would like to ask you to send the questionnaire to us by using the stamped addressed envelope.
